# Factors Influencing In-State Retention Rates of Graduating Residents From Penn State College of Medicine Compared to National Average Retention Rates

**DOI:** 10.7759/cureus.10431

**Published:** 2020-09-13

**Authors:** Melanie Patterson, Lisa Ho, Daniella Mikhail, Kevin Chiang, David R Hallan, Surav M Sakya

**Affiliations:** 1 Medicine, Penn State College of Medicine, Hershey, USA; 2 Pediatrics, Penn State College of Medicine, Hershey, USA; 3 Neurosurgery, Penn State College of Medicine, Hershey, USA

**Keywords:** physician per capita, location, residency, medical school, penn state, pennsylvania, retention rate

## Abstract

This study analyzes in-state retention rates at Penn State University (PSU) and nationally. Data were taken from the PSU handbook with location information of graduated residents and compared to data from the Association of American Medical Colleges (AAMC). The retention rate at PSU was lower than that nationally in all but three specialties. PSU retention rate was lower than that of Pennsylvania. Pennsylvania’s retention rate was lower than the national average. Community size and physician per capita may play a role in graduating resident retention rate.

## Introduction

Location is the number one priority residents consider when assessing practice opportunities [1]. This affects the geographic distribution of physicians. One approach to studying location is examining physician retention by state of training. Retention rate can allow for predictions of physician shortages [2,3].

Factors guiding retention rates include personal, family, and professional factors [4]. Quality of life, proximity to family, and quality of work environment are positive factors that influence physician retention rates. Community fit and proximity to family may reply on community size. Higher retention rates were also seen in states with fewer physicians per capita (PPC) [1].

The national retention rate is 54.6% [5]. Pennsylvania falls below this average, with a retention rate of 46.1%. When further analyzed by specialty, there are higher rates of retention in primary than specialty care [6]. Examination of physician retention at individual Pennsylvania Graduate Medical Education (GME) institutions based on medical specialties is needed. The retention rate at Penn State University (PSU) College of Medicine (Hershey, PA) has not yet been examined. Evaluating this can allow for predictions of physician shortages at this institution.

The purpose of this study is to compare residency graduate retention rates nationally with those from PSU by specialty. Considering population size and PPC are large contributors to physician retention rates, we hypothesize that the majority of retention rates based on specialty will be lower at PSU (Dauphin County PPC = 554) when compared to Pennsylvania (PPC = 337) and the national average (PPC = 276) [6].

## Materials and methods

This retrospective study used data regarding residency graduates at PSU, collected from the “Penn State College of Medicine Residency Graduate Profiles by Specialty” handbook, and national data (collected from Association of American Medical Colleges [AAMC]) [5,7]. Data were analyzed via a paired t-test with statistical significance set a p < 0.05. Graphs were created comparing in-state retention rates of graduating residents between PSU and national results. IRB exemption was granted.

## Results

Active physicians practicing in the state where they completed GME by specialty: comparison of PSU and national data

**Figure 1:**
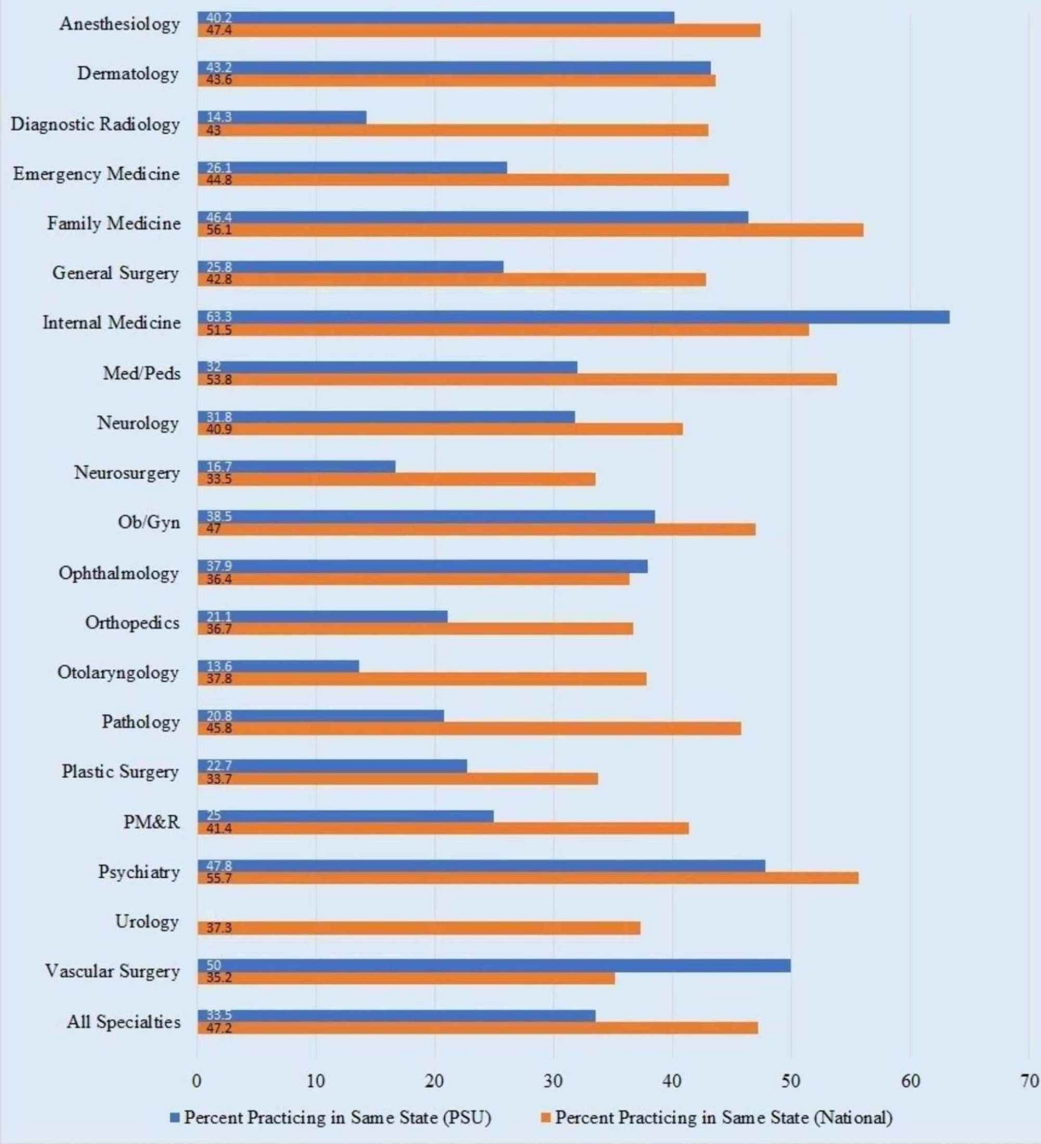
Active Physicians Practicing in the State Where They Completed GME by Specialty: Comparison of PSU and National Data. GME, Graduate Medical Education; PM&R, Physical Medicine and Rehabilitation; PSU, Penn State University

Nationally, 47.2% of graduated residents remained in the state they trained, compared to PSU’s 33.5% (Figure 1).

The program with the highest retention rate at PSU was Internal Medicine (63.3%). Nationally, Family Medicine (56.1%), Psychiatry (55.7%), and Medicine/Pediatrics (53.8%) residency programs had the highest retention rates. The retention rate of Internal Medicine residency at PSU was 11.8% higher than the national retention rate.

Urology (0%), Otolaryngology (13.6%), and Diagnostic Radiology (14.3%) residency programs had the lowest retention rate at PSU. Neurosurgery (33.5%), Plastic Surgery (33.7%), and Vascular Surgery (35.2%) residency programs had the lowest retention rate, nationally.

Compared to national data, Internal Medicine (63.3% compared to 51.5%), Ophthalmology (37.9% compared to 36.4%), and Vascular Surgery (50% compared to 35.2%) programs at PSU retained a higher percentage of graduated residents.

Physician retention in state of residency training, by state

**Figure 2:**
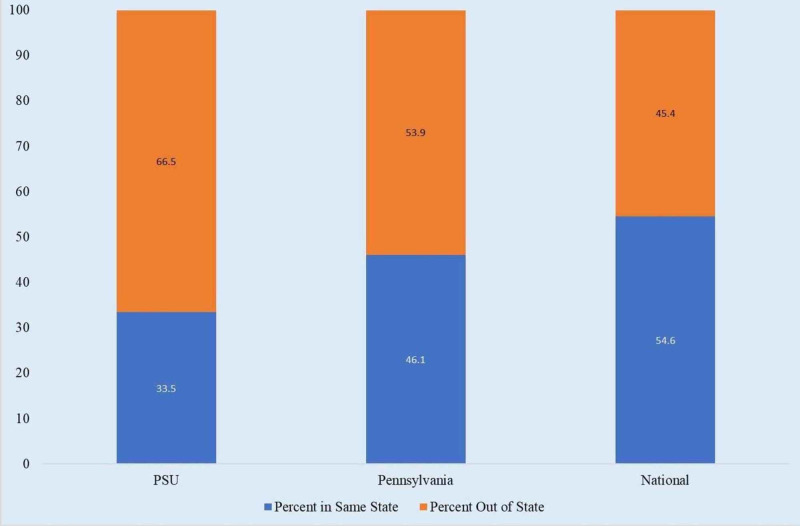
Physician Retention in State of Residency Training: Comparison of PSU and National Data. PSU, Penn State University

Approximately 54.6% of physicians practice in the same state where they completed residency (Figure 2) [5]. The retention rate in the state of Pennsylvania is 46.1%. The retention rate of PSU is 33.5%. There was a significant difference between in-state and out-of-state retention rates for both PSU (p = 0.000543) and nationally (p = 0.000567).

## Discussion

The purpose of this study was to determine the retention rate at PSU by specialty.

Internal Medicine was the specialty with the highest retention rate at PSU. Internal Medicine, a primary care specialty, may have a higher retention rate because primary care residents are more receptive to practicing in smaller communities compared to those in non-primary care specialties [1].

The specialties with the lowest retention rates at PSU are all subspecialties of medicine. Approximately 0% of residents graduating from subspecialties said that they would consider working in an area with fewer than 10,000 people [1].

When comparing the retention rates between national and PSU data, the retention rates in Internal Medicine, Ophthalmology, and Vascular Surgery were the only three specialties at PSU that exceed the retention rate reported nationally. Internal Medicine’s high retention rate is likely a reflection of primary care physicians being more likely to work in a community with a lower population [1]. Ophthalmology and Vascular Surgery had low sample sizes (28 and 4, respectively), yielding a higher retention rate.

The retention rate at PSU was lower than that nationally in all but three specialties. PSU retention rate was lower than that of Pennsylvania. Pennsylvania’s retention rate was lower than the national average.

Pennsylvania has a lower retention rate compared to the national average for all specialties. The PPC of Pennsylvania is the eighth highest out of 50 states: 320.5 per 100,000 civilians [8]. High PPC is associated with a low resident retention rate throughout the Northeast, possibly because a higher PPC is associated with increased competitiveness [8-10].

Considering PSU, location may play an additional role in the low retention rate. Location is the most important factor for residents evaluating practice opportunities [1]. Only 2% of residents would prefer to practice in a community of 25,000 people or fewer, and Hershey, PA has only 14,257 residents according to the 2010 census [8].

Limitations

This study was limited by the amount of information that was available online about national and PSU retention rates. For example, updated location was not publicly available for some graduated residents and therefore not able to be reported. The retention rate for Pediatrics was not reported due to the data regarding these residents not being fully available. Lastly, the time period in which these data were recorded from may lead to inaccurate retention rates. Job opportunities may vary over time in different locations and therefore affect retention rate.

## Conclusions

Analyzing physician in-state retention rate can help explain residents’ priorities when considering job opportunities. A large PPC and location may dissuade graduating residents from working in such an area due to high competition.
